# Genomic landscape of 340 virulent *Acinetobacter* bacteriophages reveals anti-CRISPR–enriched candidates for therapeutic prioritization

**DOI:** 10.1007/s10096-026-05580-8

**Published:** 2026-07-06

**Authors:** S. M. Iqbal Mahamud, Md. Sahim, Mahjabin Sanam, Jahin Fairuj Oishi, Jarin Tabassum, Nawshin Binte Shams, Mahfuja Maisha Ansary

**Affiliations:** 1 Department of Biological Sciences, Texas Tech University, Lubbock, TX USA; 2 Department of Biochemistry and Microbiology, North South University, Dhaka, Bangladesh

**Keywords:** *Acinetobacter baumannii*, Phage therapy, Anti-CRISPR, CRISPR spacer, Antimicrobial resistance, Carbapenem resistance

## Abstract

**Purpose:**

Carbapenem-resistant *Acinetobacter baumannii* (CRAB) represents a critical global health threat for which existing antibiotics are increasingly inadequate. This study aimed to establish a comprehensive genomic framework for the rational prioritization of virulent *Acinetobacter* bacteriophages as therapeutic candidates.

**Methods:**

We performed large-scale comparative genomic analysis of 340 virulent *Acinetobacter* bacteriophages, integrating phylogenetic reconstruction, pangenome analysis, CRISPR spacer-based host interaction mapping, Anti-CRISPR protein identification, and systematic antimicrobial resistance (AMR) gene screening.

**Results:**

Genome sizes spanned a nearly 20-fold range, with a significant negative correlation between genome size and GC content (R² = 0.139, ρ = −0.630). Phylogenetic analysis revealed extensive divergence across multiple lineages with no dominant clade. Pangenome analysis identified 20,982 unique protein families, of which 76.2% were cloud genes, confirming a highly open genome architecture. CRISPR spacer matching yielded 1,480 high-confidence matches across 100 phage genomes, providing molecular evidence of broad historical infectivity. Anti-CRISPR profiling identified *Acinetobacter* phage XC1 as an exceptional therapeutic candidate harboring 55 predicted Anti-CRISPR proteins with canonical regulatory locus architecture. AMR screening identified 21 distinct AMR gene homologs (Loose RGI hits, 22.5 to 47.1% amino acid identity) distributed heterogeneously across the dataset, confirming abundant therapeutically clean candidates while flagging a subset warranting further scrutiny before therapeutic exclusion.

**Conclusion:**

These findings provide a multi-criteria genomic framework for rational phage candidate prioritization against multidrug-resistant *Acinetobacter* infections, with direct implications for evidence-based phage therapy development.

**Supplementary Information:**

The online version contains supplementary material available at https://doi.org/10.1007/s10096-026-05580-8.

## Introduction

Antimicrobial resistance (AMR) represents one of the most pressing global health challenges of the twenty-first century, threatening the effective prevention and treatment of a wide range of bacterial infections [1, 2]. Among the most formidable contributors to this crisis is *Acinetobacter baumannii*, a Gram-negative opportunistic pathogen responsible for severe nosocomial infections including ventilator-associated pneumonia, septicemia, meningitis, and surgical wound infections, particularly among critically ill and immunocompromised patients [3, 4]. The clinical management of *A. baumannii* infections has been profoundly compromised by the rapid emergence and global dissemination of multidrug-resistant (MDR) and extensively drug resistant (XDR) strains, with carbapenem-resistant *A. baumannii* (CRAB) representing the most clinically intractable phenotype [5, 6]. In recognition of this growing threat, the World Health Organization (WHO) has consistently classified CRAB as a priority pathogen and emphasized the urgent need for the development of alternative therapeutic strategies [7, 8]. The genus *Acinetobacter* comprises approximately 82 recognized species that are widely distributed in nature and commonly found across diverse ecological niches including soil, host-associated habitats, pristine aquatic systems, and organic rich aquatic environments [9]. Beyond *A. baumannii*, several related species within the *A. calcoaceticus–baumannii* (ACB) complex, including *A. pittii*, *A. nosocomialis*, and *A. seifertii*, have also been implicated in opportunistic infections, further expanding the clinical relevance of the genus [10, 11].

The remarkable persistence of *A. baumannii* in hospital environments is largely attributed to its exceptional genomic plasticity, which facilitates the rapid acquisition and dissemination of antimicrobial resistance genes (ARGs) through horizontal gene transfer mediated by mobile genetic elements such as plasmids, transposons, and integrons [12]. This evolutionary adaptability enables the accumulation of resistance determinants targeting multiple antibiotic classes, including broad-spectrum β lactams, carbapenems, macrolides, aminoglycosides, and fluoroquinolones, progressively eroding the effectiveness of available antimicrobial therapies [13, 14]. Recent genomic studies have further highlighted the role of complex mobile genetic elements such as phage plasmids, which can function both as temperate bacteriophages and plasmids, thereby serving as reservoirs and vehicles for the dissemination of resistance genes across bacterial populations [15, 16]. These observations underscore the importance of carefully evaluating bacteriophage genomes for the presence of transmissible resistance determinants when considering phages for therapeutic applications.

As the discovery of new chemical antibiotics continues to lag behind the rapid evolution of bacterial resistance, alternative antimicrobial strategies are urgently required [17]. Bacteriophage therapy has therefore re-emerged as a promising precision medicine approach for the treatment of infections that are no longer responsive to conventional antibiotics [18]. Lytic bacteriophages possess several characteristics that make them attractive therapeutic agents. They infect and lyse bacterial hosts with high specificity, replicate at the site of infection, and employ bactericidal mechanisms that are distinct from those of traditional antibiotics [19]. Importantly, strictly lytic phages do not integrate their genomes into the host chromosome, thereby avoiding lysogeny associated risks such as the mobilization of virulence factors or antimicrobial resistance genes [20]. The clinical potential of this approach has been illustrated by successful case reports in which personalized bacteriophage cocktails were used to treat patients suffering from disseminated MDR *A. baumannii* infections, demonstrating that rigorous genomic characterization of therapeutic phages is essential for safe clinical implementation [21, 22].

Host specificity is a critical determinant of phage therapeutic utility and is primarily governed by receptor-binding structures such as tail fibers, tail spikes, and baseplate-associated proteins [23]. These proteins mediate recognition of bacterial surface receptors and frequently determine host range at the strain level. Previous studies have identified diverse tail-associated domains, including depolymerases and capsule-targeting proteins, that contribute to phage adaptation and infection specificity in *Acinetobacter* [24–26]. Understanding the diversity of these receptor-binding proteins is therefore important for predicting host range and prioritizing phages for therapeutic development. Additional case reports have shown both successful and limited outcomes of phage therapy for *A. baumannii* infections. A compassionate-use case series reported clinical improvement in several patients treated with intravenous bacteriophages for multidrug-resistant bacterial infections, including *A. baumannii*. Better outcomes were linked to proper phage selection and combination with antibiotics, while poor responses were associated with bacterial resistance and weak phage–host matching [27]. Successful clearance of XDR *A. baumannii* in sputum cultures and measurable clinical improvement within 8 days were reported in a critically ill patient treated with nebulized phage inhalation combined with antibiotics [28]. In another study, inhaled phage therapy in an elderly female patient demonstrated successful phage delivery to the infection site, with sputum Ct values decreasing from 45 to 14.7 during treatment. Phages were also detected in blood and accumulated in the intestines, providing evidence of systemic distribution following inhalation therapy [29].

The application of bacteriophage therapy must nevertheless contend with the continuous evolutionary arms race between bacteriophages and their bacterial hosts [30]. Bacteria have evolved diverse defense systems to resist viral infection, among which the CRISPR-Cas adaptive immune system plays a central role by recording fragments of invading phage DNA as spacer sequences that guide subsequent immune responses against matching phages [31]. These CRISPR spacer sequences therefore represent valuable molecular records of historical phage host interactions. In response, bacteriophages have evolved counter defense mechanisms such as Anti-CRISPR (Acr) proteins, which inhibit CRISPR-Cas activity and enable successful infection [32]. Anti-CRISPR genes are frequently associated with conserved transcriptional regulators and clustered within phage genomes alongside other anti defense modules, allowing their identification through genomic context-based approaches such as guilt-by-association analyses [33]. Although the CRISPR-Cas system is only one of several bacterial defense mechanisms against bacteriophages, other systems have also been identified, including restriction–modification systems, abortive infection systems, and more recently characterized mechanisms such as CBASS, Thoeris, and Gabija [34, 35]. In the present study, we focused specifically on CRISPR-Cas because of its relative prevalence in *A. baumannii* and its extensive characterization. A large-scale analysis of 4,977 *A. baumannii* RefSeq genomes showed that approximately 13.84% of isolates harbor functional CRISPR-Cas systems [36]. This prevalence makes CRISPR-Cas the most extensively characterized adaptive immune system identified in this pathogen. Also, CRISPR spacers represent long-term genomic records of historical phage infection events and are directly exploitable from sequence data alone without requiring experimental characterization of each defense system [37].

Despite the increasing availability of sequenced bacteriophage genomes, comprehensive comparative analyses of *Acinetobacter* phages remain relatively limited. Early studies examined only a small number of genomes, while more recent analyses expanded the dataset to improve clustering, taxonomic resolution, and understanding of genomic organization [38–40]. However, these investigations primarily focused on genomic diversity and classification, with comparatively limited emphasis on evaluating phage genomes in the context of therapeutic potential or their suitability for phage therapy against antibiotic resistant *Acinetobacter* infections. Large-scale genomic studies are therefore essential for establishing systematic frameworks that can guide the identification and prioritization of phages with potential therapeutic value.

In this study, we conducted a large-scale comparative genomic analysis of 340 virulent *Acinetobacter* bacteriophages to establish a systematic framework for evaluating genomic features relevant to therapeutic prioritization against multidrug-resistant *A. baumannii*. We integrated phylogenetic reconstruction, pangenome analysis, CRISPR spacer-based host interaction inference, Anti-CRISPR candidate identification, and antimicrobial resistance gene screening to evaluate diversity, host interaction history, and genomic safety. Together, these analyses provide a multi-criteria genomic framework for the rational prioritization of virulent *Acinetobacter* phages for future therapeutic development.

## Materials and methods

### Genome selection and lifestyle classification

**Fig. 1:**
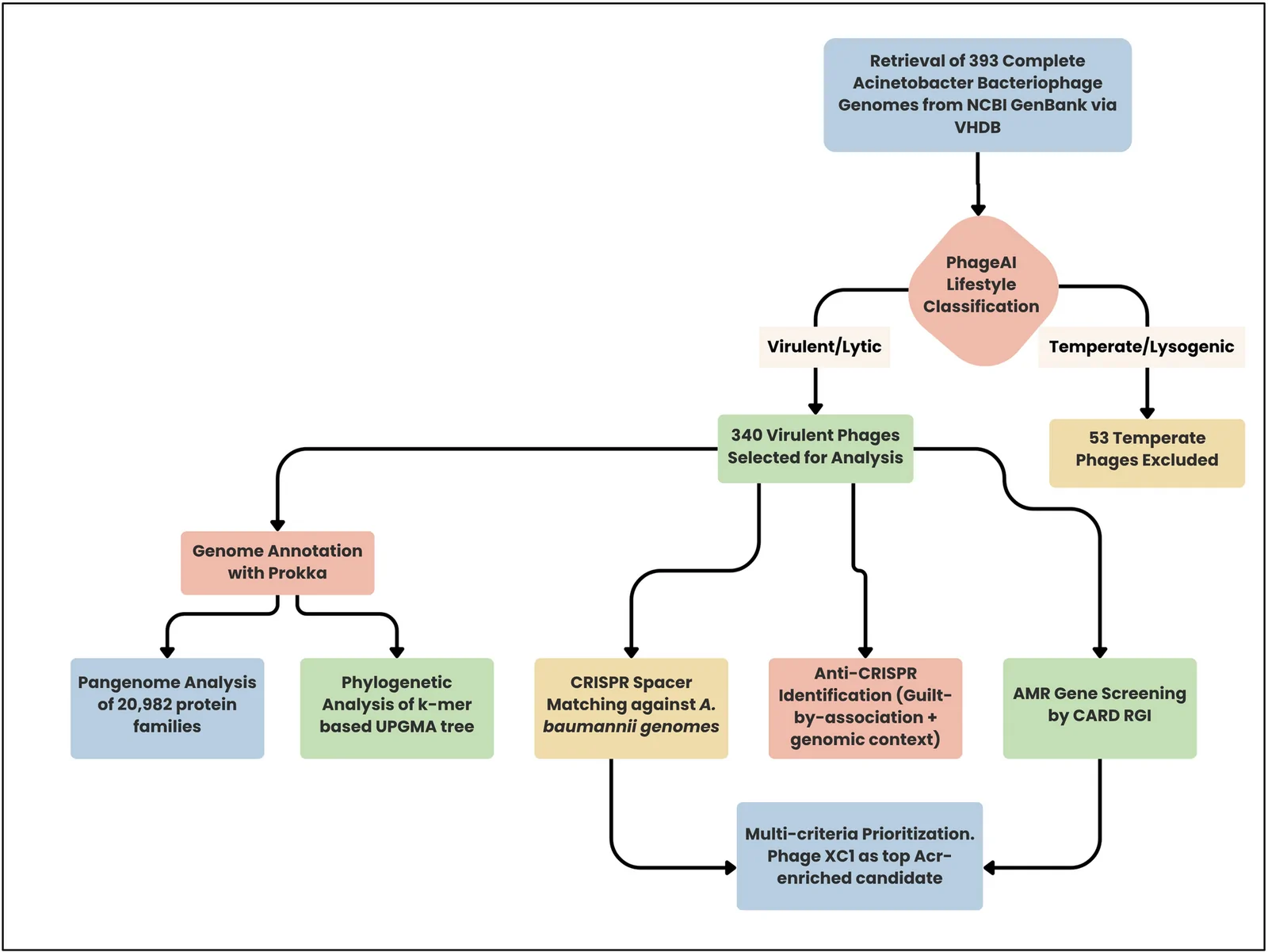
Workflow for genome selection, filtering, lifestyle classification, and downstream analyses of *Acinetobacter* bacteriophages

A total of 393 complete *Acinetobacter* bacteriophage genomes were retrieved from the NCBI GenBank database [41] using accession numbers obtained from the Virus-Host Database (VHDB) [42], queried in October 2025. Only fully assembled genome sequences were retained; incomplete assemblies and plasmid-derived sequences were excluded. The infection lifestyle of each phage was predicted using PhageAI [43], a machine learning platform for bacteriophage lifestyle prediction, which classified 340 genomes as virulent (lytic) and 53 as temperate (lysogenic). All downstream analyses were restricted to the 340 virulent phages. Genome size and GC content were obtained from GenBank records, and host association metadata were retrieved from VHDB annotations. A complete overview of genome characteristics is provided in Supplementary Table S1. A flowchart summarizing the full genome selection and exclusion process, and the downstream analyses performed on the final dataset, is provided as Fig. 1.

### Genome annotation and pangenome analysis

Protein-coding sequences across all 340 virulent phage genomes were annotated using Prokka [44], implemented through the European Galaxy server [45]. The predicted protein sequences were aggregated into a unified dataset and grouped using a custom Python script (publicly available in the project repository; Code Availability section) that collapsed identical amino-acid sequences into non-redundant protein families. Each unique sequence was treated as a protein family, and its occurrence across genomes was recorded to generate a frequency table. Based on their distribution across genomes, protein families were classified into three categories following standard pangenome definitions: cloud (detected in only a single genome), shell (detected in 2 to 24 genomes), and persistent (detected in ≥ 25 genomes). Because clustering was performed using exact amino-acid sequence identity, a universal core genome would correspond to protein families present in all 340 analyzed genomes at 100% identity. This strict sequence-identity criterion provides a conservative estimate of sequence-level conservation across genomes.

To further evaluate pangenome openness, a pangenome accumulation curve was generated by sequentially adding genomes across 100 random permutations and calculating the cumulative number of unique protein families based on exact amino acid sequence identity. The mean cumulative pangenome size and standard deviation were calculated across all permutations and plotted as a function of the number of genomes included.

### Phylogenetic analysis

Pairwise genomic distances among the 340 virulent phage genomes were calculated using an alignment-free k-mer frequency–based comparison of nucleotide sequences (k = 13), implemented in a custom Python script (available in the project repository; see Code Availability section). A moderate k-mer size (k = 13) was selected to balance discriminatory resolution and computational tractability for large-scale phage genome comparison. The resulting distance matrix was used to infer a whole-genome phylogenetic tree using the Unweighted Pair Group Method with Arithmetic Mean (UPGMA). The annotated phylogeny was visualized using Interactive Tree of Life (iTOL) v6 [46].

### Functional and host-interaction profiling

#### CRISPR spacer-based host range analysis

CRISPR spacer sequences were extracted from 350 fully assembled and complete *Acinetobacter baumannii* genomes retrieved from NCBI GenBank (2025) using CRISPRCasFinder [47] with default parameters. Draft assemblies, contigs, and scaffold-level genomes were excluded to ensure reliable spacer identification. A total of 508 unique spacer sequences were identified and used for downstream analysis. A nucleotide database of all 340 phage genomes was constructed using BLAST+ v2.12.0 [48], and spacer sequences were aligned against it using BLASTn optimized for short queries. Alignments were retained at a minimum of 95% nucleotide identity and 25 bp alignment length. The number of high-confidence spacer matches per phage genome was calculated as an indicator of CRISPR-supported host association. Spacer-based matches were interpreted as evidence of prior phage–host interactions and potential host associations rather than definitive confirmation of host range or active CRISPR-Cas immunity.

#### In silico identification of anti-CRISPR candidates

Putative Anti-CRISPR (Acr) proteins were identified across the 340 phage genomes using a genomic context-based guilt-by-association strategy [49]. Candidate proteins were prioritized based on their proximity to Anti-CRISPR-associated (*Aca*) proteins or Aca-like transcriptional regulators, which are commonly associated with anti-defense loci. To integrate these signals, a composite support score (maximum = 3.0) was calculated by assigning one point each for protein size within the known Acr range, regulatory gene proximity, and cross-genome recurrence, and candidates were ranked accordingly.

#### Antimicrobial resistance gene detection

Putative AMR gene homologs encoded within phage genomes were identified using the Resistance Gene Identifier (RGI) tool against the Comprehensive Antibiotic Resistance Database (CARD, version 3.2.7) [50], including Loose hits that fall below CARD’s curated bitscore cutoff for each gene family. Percentage amino acid identity to the corresponding CARD reference sequence was recorded for every detection. A presence-absence matrix was constructed and visualized as a heatmap, with cell color scaled to amino acid identity.

To assess whether *dfrA* homologs were associated with integron-mediated mobilization, all phage genomes containing at least one *dfrA* homolog (Loose RGI hit) were analyzed using IntegronFinder v2.0.6 [51] in sensitive mode (--local-max). The tool was used to identify integron integrases (*intI*), phage integrases, and attC recombination sites. Based on the detected features, genomes were classified as complete integrons (*intI* + attC), CALIN elements (attC only), or In0 elements (integrase only).

### Statistical analysis

The association between genome size and GC content was evaluated using linear regression and independently validated using Spearman rank correlation. All statistical analyses were performed in Python v3.9 using custom analysis scripts deposited in a public repository (Code Availability section), and statistical significance was defined at a threshold of 0.05.

## Results

### Dataset composition and genome size distribution

Of the 393 complete *Acinetobacter* phage genomes retrieved, PhageAI classified 340 (86.51%) as virulent and 53 (13.49%) as temperate. The 340 virulent phages exhibited substantial diversity in genome architecture and nucleotide composition **(**Supplementary Table S1**)**. Genome sizes ranged from 15.8 kb to 286.4 kb with a median of 45.2 kb, reflecting a nearly 20-fold range that suggests markedly different genomic strategies across lineages. GC content ranged from 30.1% to 51.6% with a median of 39.4%, indicating broad compositional heterogeneity across the phage population.

### Genome size and gc content show a significant negative association

**Fig. 2:**
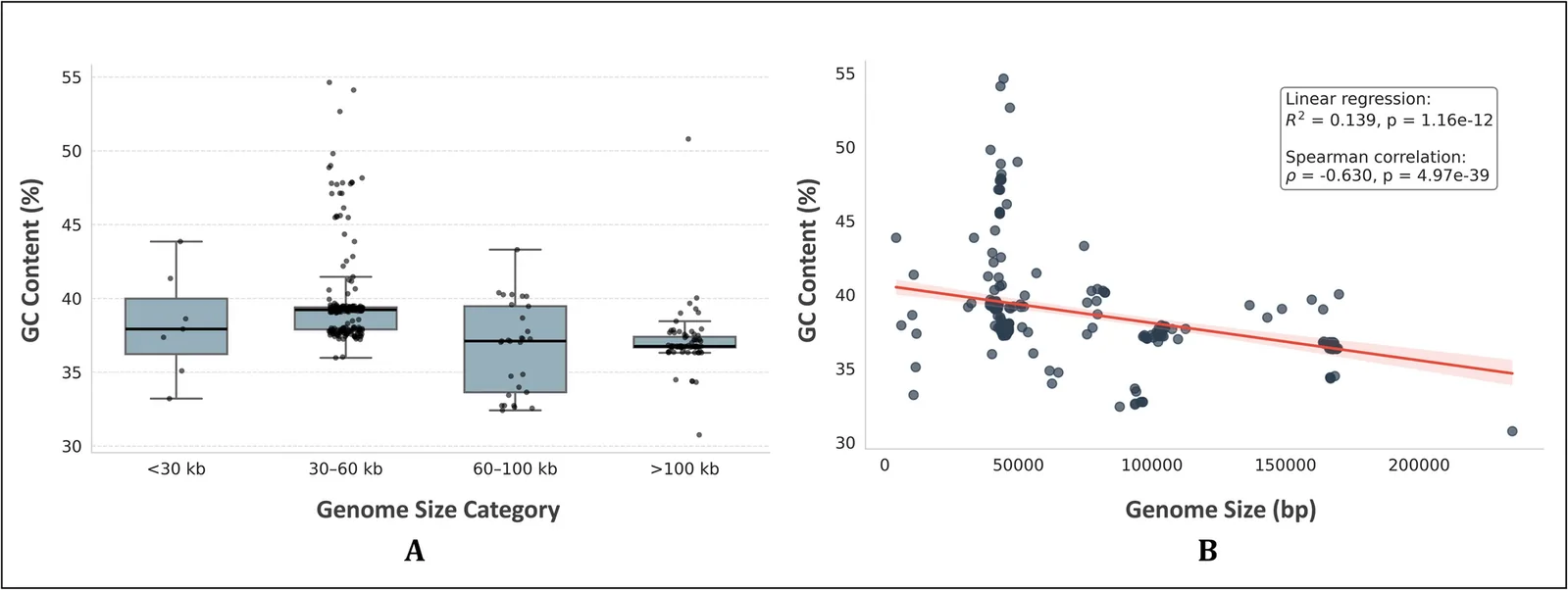
**(A)** Boxplot distribution of GC content across four genome size categories (less than 30 kb, 30 to 60 kb, 60 to 100 kb, and greater than 100 kb). (**B**) Linear regression (R² = 0.139, *p* = 1.16 × 10⁻¹²) and Spearman rank correlation (ρ = −0.630, *p* = 4.97 × 10⁻³⁹) demonstrating the negative association between genome size and GC content among virulent *Acinetobacter* phages

Analysis of GC content across genome size categories revealed a systematic trend in which smaller phage genomes displayed higher median GC content and greater variability, while larger genomes exceeding 100 kb showed lower and more narrowly distributed GC content values (Fig. 2A). Considerable overlap was nonetheless observed across size groups, suggesting that factors beyond genome size contribute to nucleotide composition.

Linear regression confirmed a statistically significant negative association between genome size and GC content (R² = 0.139, *p* = 1.16 × 10⁻¹²; Fig. 2B), rejecting the null hypothesis of no association. The low R² value indicates that genome size accounts for only 13.9% of GC content variance. Spearman rank correlation independently confirmed a strong monotonic negative relationship (ρ = −0.630, *p* = 4.97 × 10⁻³⁹), supporting the robustness of this finding across the full-size range. Together, these results demonstrate that while GC content tends to decrease with increasing genome size, additional evolutionary pressures including host adaptation, replication strategy, and horizontal gene transfer most likely shape the residual compositional variation.

### Phylogenetic analysis reveals extensive genomic diversity across virulent *Acinetobacter* phages

**Fig. 3:**
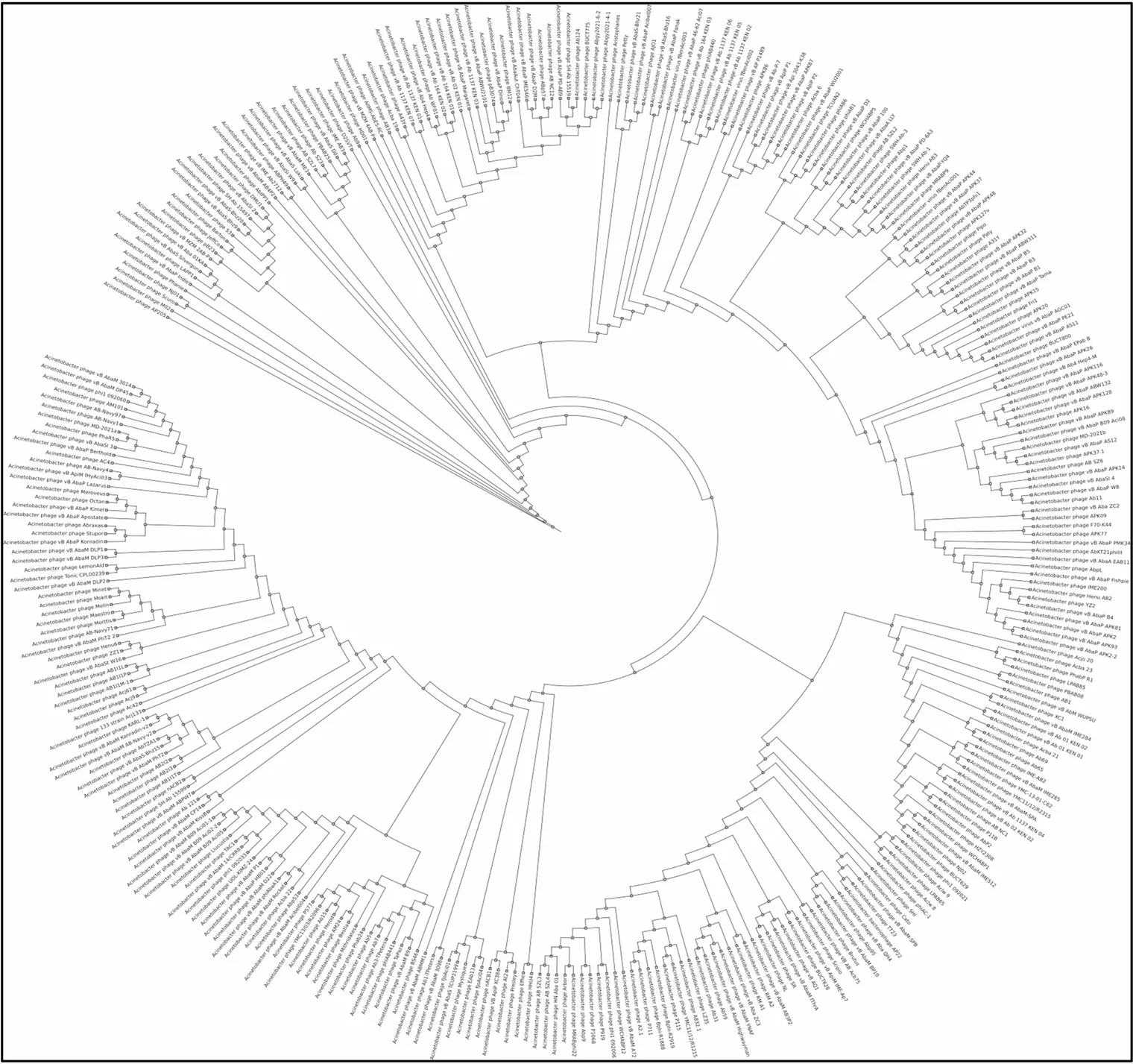
Whole-genome UPGMA phylogenetic tree of 340 virulent *Acinetobacter* bacteriophages based on pairwise k-mer distances (k = 13)

Whole-genome phylogenetic analysis of the 340 virulent phages revealed substantial genomic diversity distributed across multiple well-separated lineages **(**Fig. 3**).** The inferred tree displayed a highly heterogeneous topology, with closely related phages forming compact subclades and more divergent genomes separated by longer branch lengths, reflecting a wide spectrum of evolutionary distances within the dataset. A subset of phages clustered into dense, well-resolved subclades indicative of recent common ancestry, while a substantial proportion of genomes occupied isolated phylogenetic positions characterized by long terminal branches, consistent with independent evolutionary trajectories and lineage-specific divergence. No single dominant clade encompassed the majority of the dataset, underscoring the breadth of genomic heterogeneity within the virulent *Acinetobacter* phage population. Overall, the phylogenetic reconstruction captures both the deep evolutionary divergence across major lineages and the localized conservation within specific subclades, providing a structural framework for the selection of phylogenetically representative candidates for rational phage cocktail design.

### Pangenome analysis reveals an open genome architecture with no conserved core

**Fig. 4:**
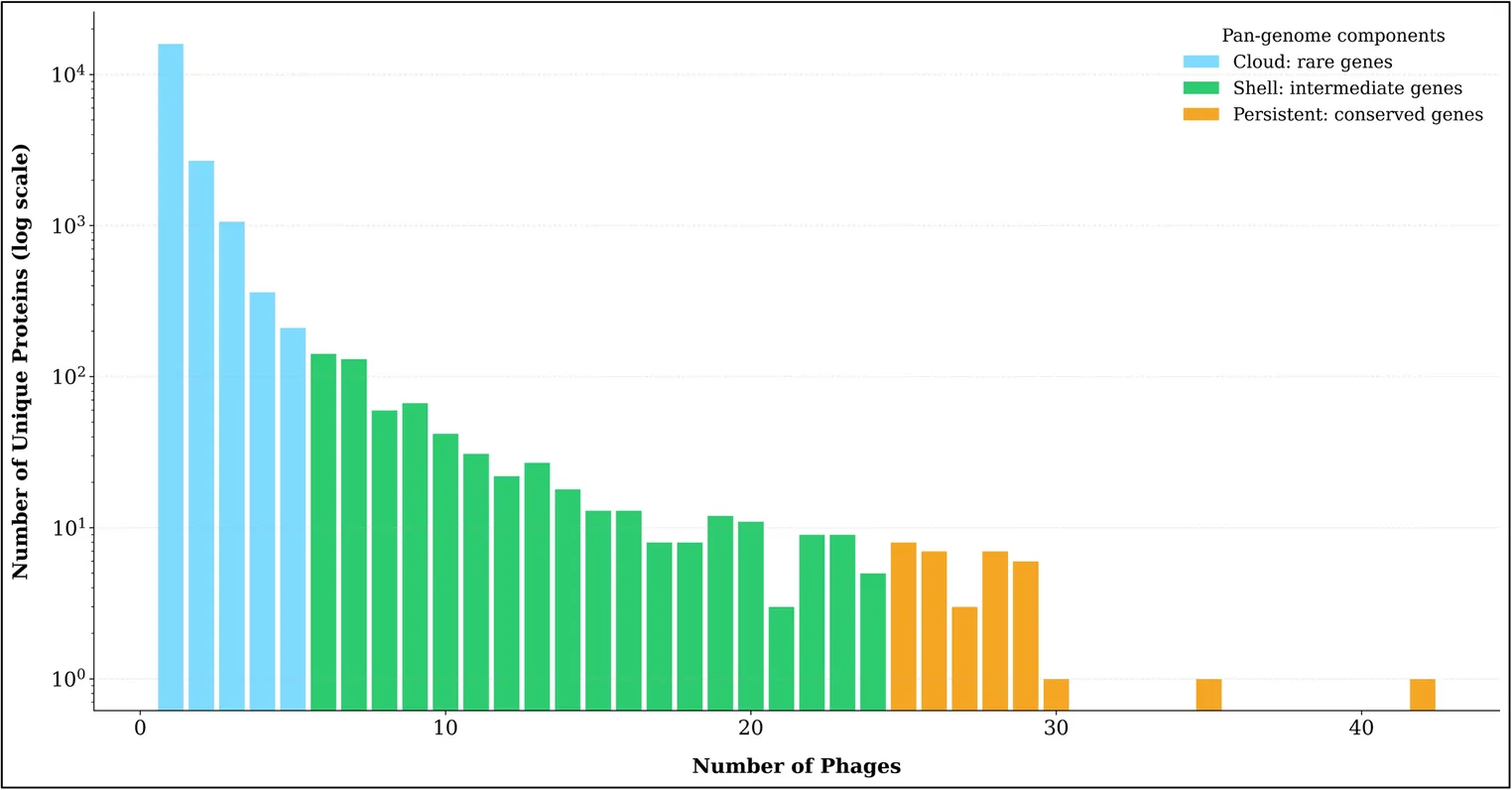
Pangenome frequency distribution of 340 virulent *Acinetobacter* phage genomes. The x-axis represents the number of phage genomes in which each protein family was detected, and the y-axis represents the number of unique protein families. Blue = cloud genes, green = shell genes, orange = persistent genes

Pangenome analysis of the 340 virulent phage genomes yielded 20,982 unique protein families, with occurrence frequencies spanning from a single genome to a maximum of 42 genomes (12.4% of the dataset), indicative of extensive gene content diversity across the dataset **(**Fig. 4; Supplementary Table S2). The large majority of protein families (15,983; 76.2%) belonged to the cloud genome, each detected in only a single phage genome, reflecting a large reservoir of rare and lineage-specific gene content. Shell-class protein families were shared across a moderate subset of related phages, suggesting partial conservation within specific lineages. The persistent genome comprised a comparatively small fraction of broadly distributed proteins, likely representing conserved structural or replication-associated functions.

The absence of a universal core genome likely reflects the extremely stringent clustering threshold (100% amino-acid identity), which captures only perfectly conserved sequences. Collectively, these findings establish that the virulent *Acinetobacter* phage pangenome is highly open and dominated by accessory gene content, suggesting that horizontal gene transfer and modular recombination are likely important contributors to genomic diversity within this phage population.

**Fig. 5:**
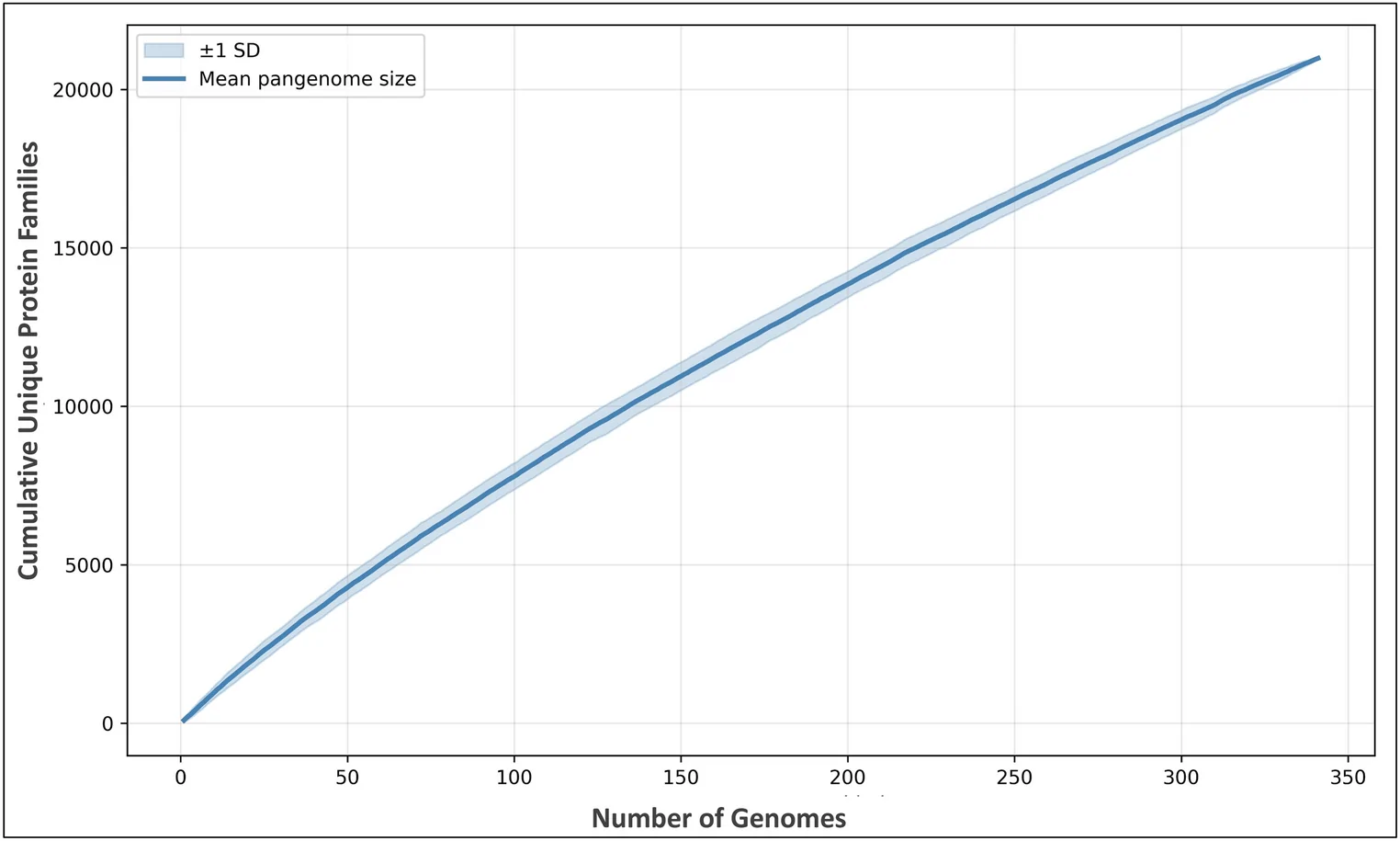
Pangenome accumulation curve of 340 virulent *Acinetobacter* phages. The cumulative number of unique protein families was calculated by sequentially adding genomes across 100 random permutations. The solid line represents the mean cumulative pangenome size, and the shaded region indicates ± 1 standard deviation. The continued increase in protein family richness without evidence of saturation supports an open pangenome architecture

To further assess pangenome openness, a pangenome accumulation analysis was performed using 100 random genome-addition permutations (Fig. 5). The cumulative number of unique protein families increased continuously with the addition of new genomes and reached 20,982 protein families after inclusion of all 340 phages. The absence of a plateau indicates that new genomes continue to contribute previously unobserved protein families, supporting the conclusion that the *Acinetobacter* phage pangenome remains highly open.

### Frequency analysis identifies conserved baseplate and lysis-associated proteins

**Fig. 6:**
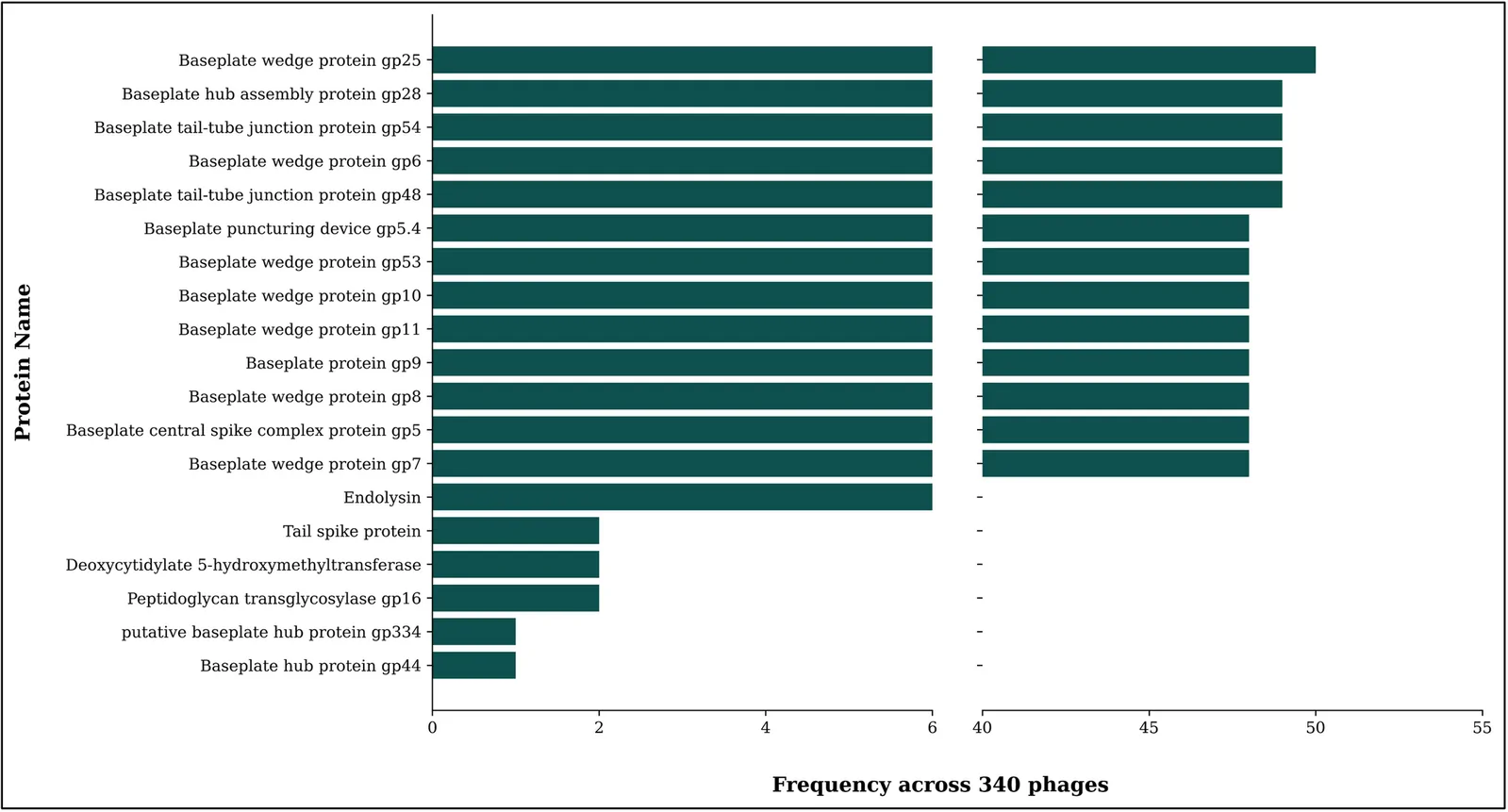
Frequency distribution of therapeutically relevant structural and functional proteins across 340 virulent *Acinetobacter* phage genomes. The x-axis is broken to illustrate the two distinct frequency ranges observed among the selected proteins

Frequency-based analysis of the 34,299 predicted proteins (representing the total raw annotation output prior to clustering into 20,982 non-redundant protein families during pangenome analysis) across 340 *Acinetobacter* phage genomes was conducted with emphasis on proteins with known or predicted roles in host recognition, cell lysis, and infection specificity, given the therapeutic focus of this study. The complete frequency distribution of all annotated proteins is provided in Supplementary Table S3. Among these categories, baseplate-associated components were the most recurrently detected across the dataset (Fig. 6). The most frequently observed proteins included baseplate wedge proteins gp25, gp6, gp53, gp7, gp8, gp10, and gp11, baseplate hub assembly protein gp28, baseplate tail-tube junction proteins gp48 and gp54, baseplate central spike complex protein gp5, and baseplate puncturing device detected in 48–50 phage genomes. The consistent prevalence of these components indicates strong evolutionary conservation ofrelease. Tail spike protein, peptidoglycan transglycosylase the baseplate infection module among *Acinetobacter* phages.

Among proteins involved in host cell lysis, endolysin was the most frequently detected, occurring in six phage genomes and highlighting its conserved role in host cell wall degradation and phage progeny release. Tail spike protein, peptidoglycan transglycosylase gp16, and deoxycytidylate 5-hydroxymethyltransferase were each identified in two phage genomes, suggesting specialized functions in host surface recognition and DNA modification. Putative baseplate hub proteins gp343 and gp44 were detected in only a single phage genome each, potentially reflecting lineage-specific or modular accessory components. Overall, the annotation profile indicates that *Acinetobacter* phages encode a diverse set of proteins associated with host interaction and lytic processes, while the presence of auxiliary enzymatic functions suggests additional layers of host adaptation across the phage population.

### CRISPR Spacer matching identifies phages with evidence of extensive historical host interactions

Alignment of *A. baumannii* CRISPR spacer sequences against the 340 phage genomes identified 1,480 high-confidence spacer–phage matches across 100 phages (29.4% of the analyzed dataset). This finding indicates that nearly one-third of the virulent phage population carries genomic evidence of prior infection of *A. baumannii* hosts, as recorded in the CRISPR adaptive immune memory of clinical and environmental strains.

**Fig. 7:**
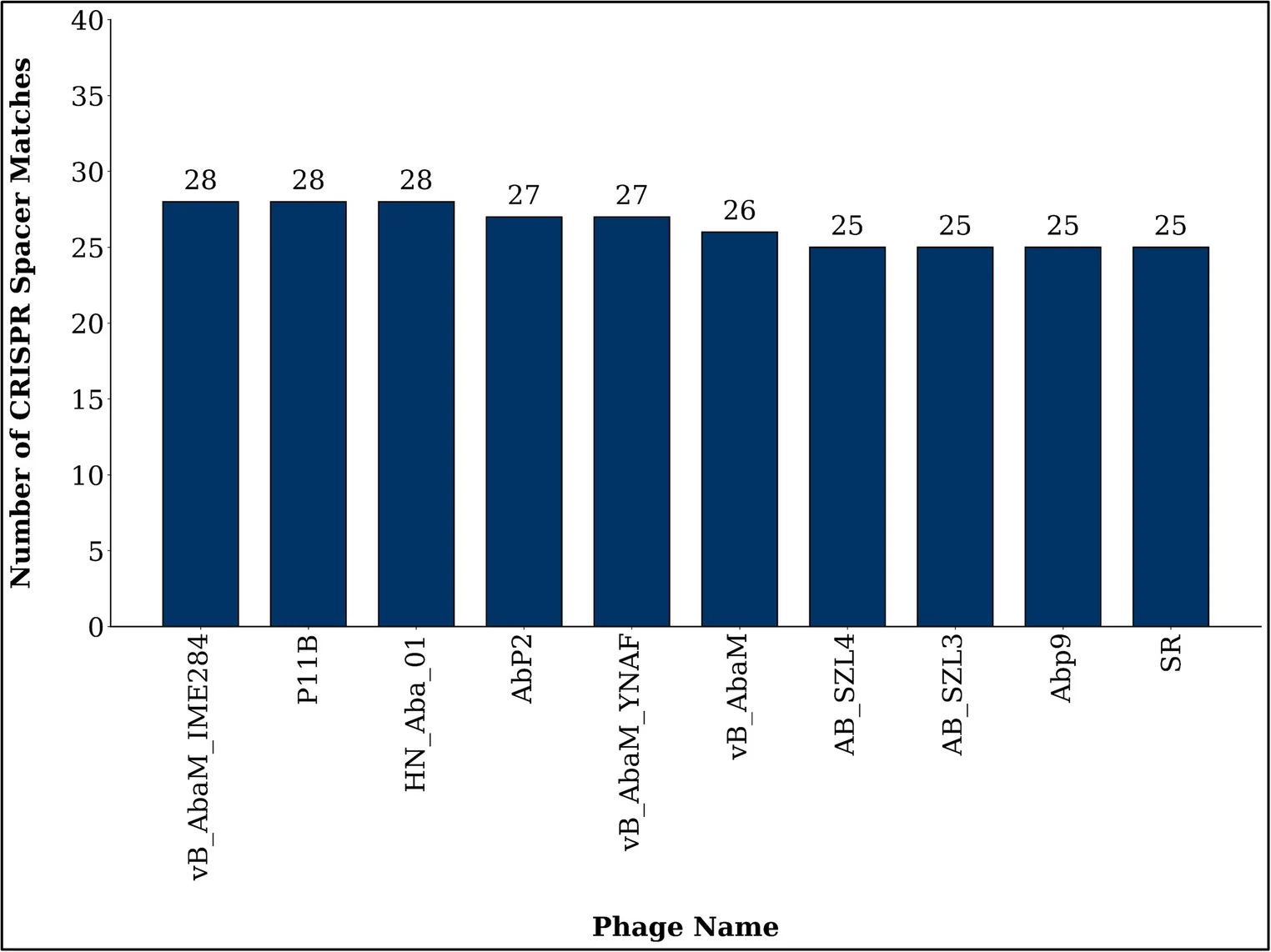
Distribution of CRISPR spacer matches among *Acinetobacter* phages with high host association (displaying phages with 25 or more independent spacer hits). Each spacer represents a distinct historical *A. baumannii* infection interaction

The abundance of spacer matches varied markedly among phage genomes, reflecting heterogeneity in host exposure and interaction frequency (Fig. 7). Multiple phages accumulated more than 25 independent spacer matches, suggesting repeated encounters with *A. baumannii* across diverse strains or populations. Because each independent spacer represents a distinct historical infection interaction, high numbers of spacer matches suggest repeated interactions with *A. baumannii* hosts and potential broad host exposure, a property of particular relevance for therapeutic applications. In contrast, phages with few or no spacer matches may reflect narrower host ranges, infection of CRISPR-deficient strains, or limited representation of their target hosts in current genome databases. Figure 7 displays phages with the highest spacer abundance (≥ 25 matches), while Supplementary Table S4 provides complete matching data for all 100 phages exhibiting CRISPR spacer associations.

### Identification of Anti-CRISPR enriched phages for therapeutic prioritization

**Fig. 8:**
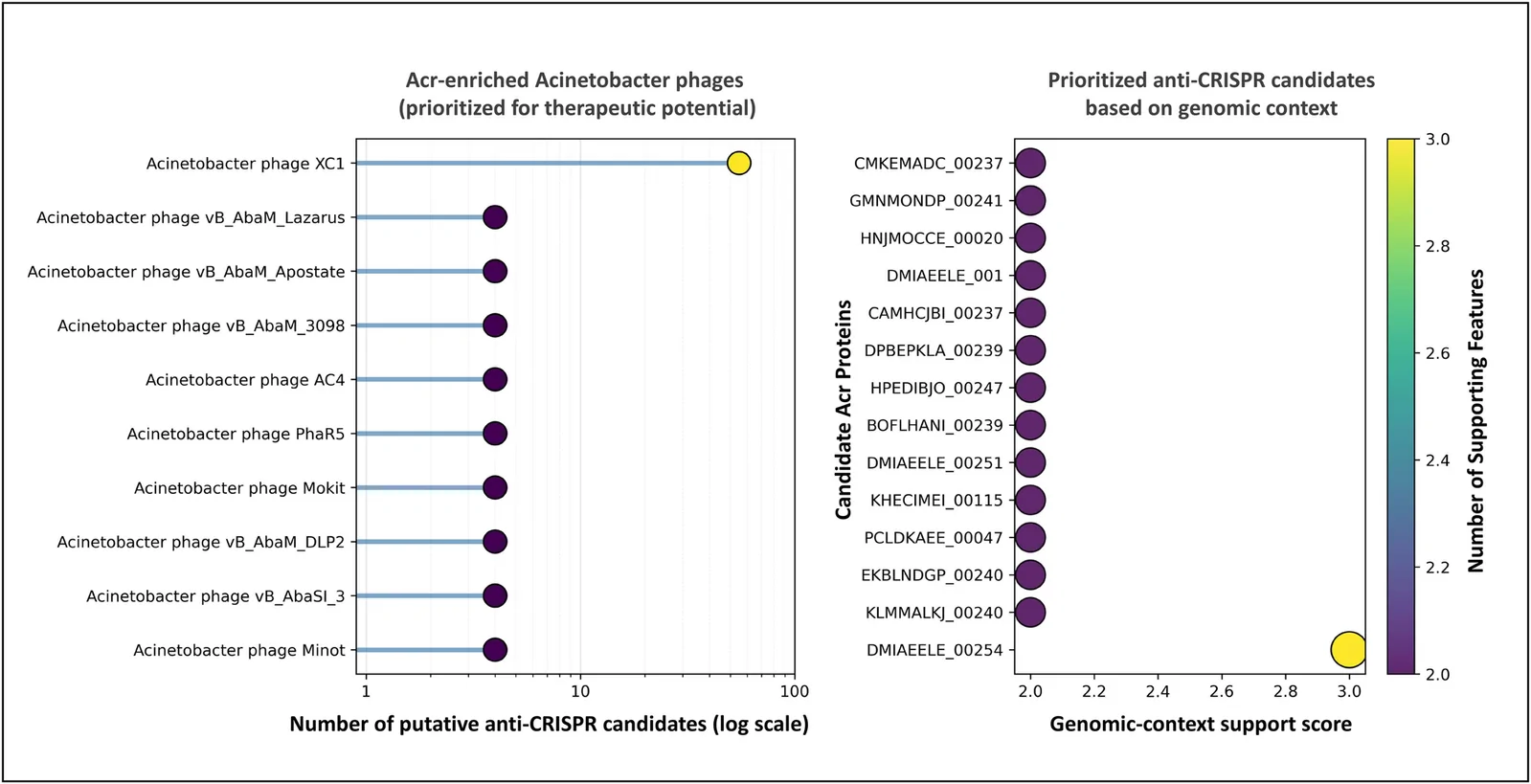
Identification of Acr-enriched *Acinetobacter* phages. (**A**) Acr candidate distribution across phage genomes. Phages are ranked by number of predicted Acr proteins (log scale). (**B**) Genomic-context support scores for prioritized Acr candidates. Higher scores indicate stronger evidence for Acr function based on genomic architecture

Application of the guilt-by-association Anti-CRISPR identification pipeline revealed a highly skewed distribution of predicted Acr candidates across the analyzed phage genomes **(**Fig. 8A**)**. Most phages encoded between one and four putative Acr proteins. In contrast, *Acinetobacter* phage XC1 emerged as a clear outlier, harboring 55 predicted Acr candidates, which represents more than an order of magnitude greater density than any other phage in the dataset. Several additional phages, including vB_AbaM_Lazarus, vB_AbaM_Apostate, vB_AbaM_3098, AC4, PhaR5, Mokit, vB_AbaM_DLP2, vB_AbaSI_3, and Minot, exhibited moderate enrichment with approximately four predicted candidates each. However, none approached the extensive Acr repertoire observed in *Acinetobacter* phage XC1.

Genomic context analysis further supported these predictions **(**Fig. 8B). The prioritized candidates consisted predominantly of small hypothetical proteins ranging from 74 to 78 amino acids, consistent with the typical size range reported for known Acr proteins. These proteins were frequently located adjacent to Gp33-like transcriptional regulators, forming genomic arrangements consistent with previously described Acr regulatory loci. For example, *Acinetobacter* phage Minot displayed a canonical organization, with candidate protein BHJCOJBD_00242 positioned immediately downstream of the predicted regulator BHJCOJBD_00240. In contrast, *Acinetobacter* phage XC1 contained a contiguous cluster of twelve small hypothetical proteins (PCCACAJL_00003 to PCCACAJL_00022) within a single genomic region. Among all candidates, DMIAEELE_00254 from *Acinetobacter* phage XC1 achieved the highest genomic-context support score of 3.0, indicating strong evidence based on protein size, regulatory proximity, and cluster organization. These observations identify *Acinetobacter* phage XC1 as a high-priority candidate for future experimental evaluation. Complete Acr candidate annotations, genomic coordinates, and support scores for all analyzed phages are provided in Supplementary Table S5. Visualization of candidate distribution within representative Acr-enriched phages showed that predicted Anti-CRISPR proteins were not evenly dispersed throughout the annotated gene order, but instead frequently occurred in localized clusters (Supplementary Figure S1). Such clustering is consistent with the genomic-context–based strategy used for candidate identification and suggests that candidate Acr loci may be concentrated within specific genomic regions of certain phages.

### Antimicrobial Resistance Gene Distribution Across Phage Genomes

**Fig. 9:**
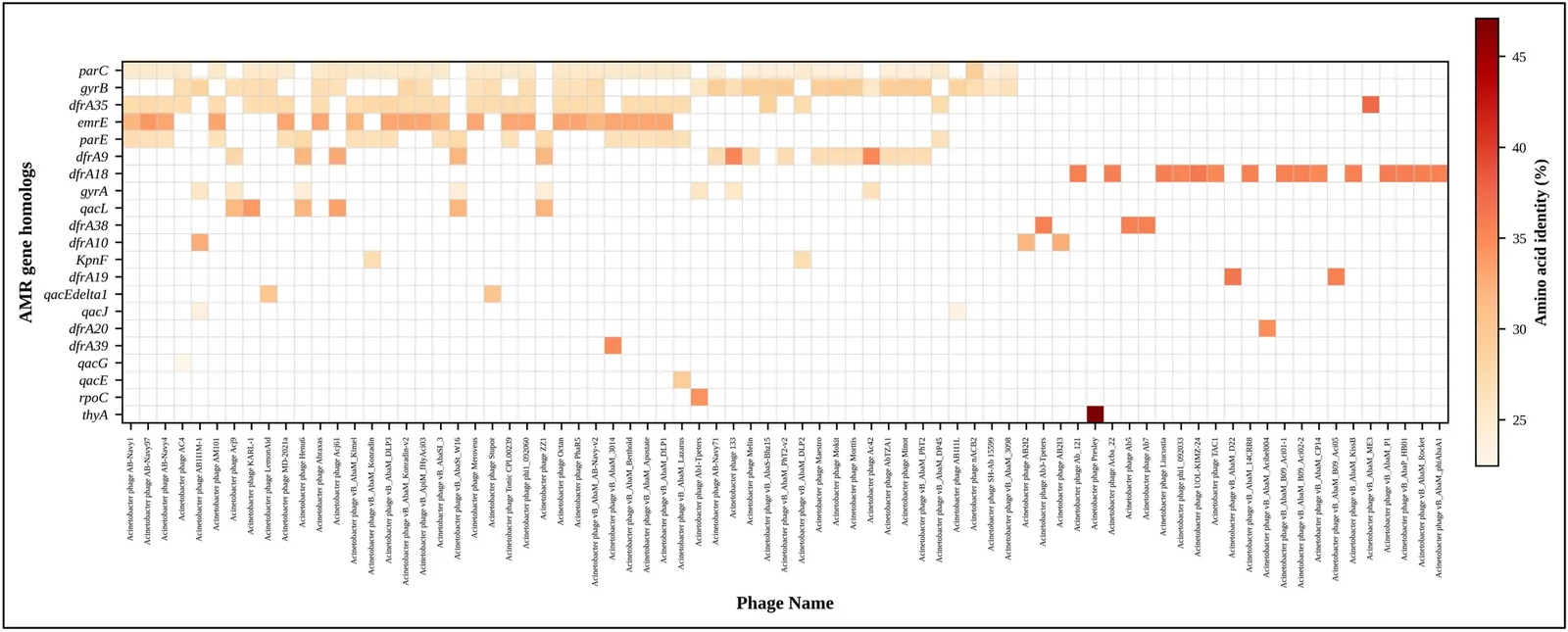
Heatmap of AMR gene homologs detected across virulent *Acinetobacter* phage genomes via Loose RGI hits against the CARD database. Cell color intensity reflects percentage amino acid identity to the corresponding CARD reference sequence; white indicates absence of the gene

Systematic screening of all 340 phage genomes using the CARD Resistance Gene Identifier (RGI) revealed a heterogeneous distribution of AMR gene homologs across the dataset (Fig. 9). In total, 21 distinct gene families were detected as Loose RGI hits, with amino acid identity to CARD reference sequences ranging from 22.5 to 47.1%, representing genes linked to antibiotic target modification, efflux systems, and metabolic pathway alterations.

Genes associated with quinolone resistance and folate pathway inhibition were among the most frequently detected features. Sequences annotated as DNA gyrase subunits (*gyrA* and *gyrB*) and topoisomerase IV subunits (*parC* and *parE*) were identified in a considerable fraction of phage genomes. These genes may represent host-derived housekeeping elements captured during phage evolution rather than functional resistance determinants actively disseminated by phages. Members of the *dfrA* family of dihydrofolate reductase genes, including *dfrA35*,* dfrA20*, and *dfrA38*, were also detected across several phage genomes, suggesting potential associations with folate pathway related resistance mechanisms.

A total of 71 phage genomes carried at least one *dfrA* gene homolog, across eight distinct alleles (*dfrA9*,* dfrA10*,* dfrA18*,* dfrA19*,* dfrA20*,* dfrA35*,* dfrA38*,* and dfrA39*). All 71 genomes were screened for integron architecture using IntegronFinder. No integrase gene (integron-specific or general phage) was detected in any genome, and no complete integron, CALIN element, or In0 element was identified (Supplementary Table S6). Twenty-nine genomes (40.8%) showed a single weak attC-like match, but these were isolated, not clustered into cassette arrays, and not located near any integrase gene, consistent with background noise rather than true attC sites. Efflux associated genes were less frequent but showed scattered occurrence across the dataset. These included *emrE*, *KpnF*, and several members of the *qac* gene family (*qacE*, *qacEdelta1*, *qacG*, *qacJ*, and *qacL*), which are commonly linked to multidrug or disinfectant resistance.

The heatmap illustrates substantial variability in AMR gene homolog content among phage genomes. While some phages carried multiple homolog loci, the majority encoded few or none. Given the low identity of these detections to curated CARD references, they should be interpreted as candidate sequences rather than confirmed resistance determinants. These findings nonetheless highlight the importance of systematic genomic safety screening when selecting phages for therapeutic applications, since confirming or excluding functional resistance activity in flagged phages requires additional sequence or experimental validation.

## Discussion

The 340 virulent phage genomes exhibited substantial diversity in genome size, spanning from 15.8 kb to 286.4 kb, alongside a broad GC content range of 30.1% to 51.6%. Statistical analysis confirmed a significant negative association between genome size and GC content (R² = 0.139, ρ = −0.630, *p* = 4.97 × 10⁻³⁹), indicating that larger phage genomes tend toward lower GC values. However, the low R² value demonstrates that genome size accounts for only approximately 14% of GC content variance, strongly suggesting that additional evolutionary forces independently govern nucleotide composition. Similar findings have been reported in comparative genomic analyses of bacteriophages infecting other opportunistic Gram-negative pathogens. These forces likely include host-imposed codon usage pressures, the compositional signatures of horizontally acquired genetic material, and replication strategy constraints. This observation aligns with recent genomic characterizations of individual *Acinetobacter* phages, where genome size and GC content consistently appear to vary independently of one another across diverse phage types [39, 52, 53]. A comprehensive survey of 132 *A. baumannii* phages similarly noted genome sizes ranging from 4 to 234 kb with no consistent compositional pattern across size classes, reinforcing the conclusion that these two properties must be treated as independent axes of genomic diversity rather than correlated outputs of a single evolutionary process [54]. The considerable variability in GC content among smaller phage genomes, compared to the narrower distribution observed in phages exceeding 100 kb, further suggests that small *Acinetobacter* phages are subject to a more heterogeneous set of evolutionary pressures, possibly reflecting greater diversity in their ecological origins and host interaction histories. Understanding these compositional patterns is important for both phage classification and therapeutic applications. GC content mismatches between phage and host genomes can influence translational efficiency and replication kinetics in ways that have direct consequences for infection outcomes [55, 56].

Whole-genome phylogenetic analysis revealed extensive genomic heterogeneity across the 340 virulent phages, with no single dominant lineage encompassing the majority of the dataset. The tree displayed a highly heterogeneous topology, with closely related phages forming compact subclades while more divergent genomes were separated by long branch lengths, reflecting a wide spectrum of evolutionary distances. This mixed architecture is consistent with the mosaic model of phage evolution, in which functional genomic modules are exchanged among distantly related phages through recombination events, generating novel genomic configurations without necessitating complete phylogenetic separation [57]. The arms race between *A. baumannii* and its phages, encompassing bacterial defenses including CRISPR-Cas, restriction-modification, and abortive infection systems alongside phage counter-adaptations, continuously drives evolutionary diversification and discourages the emergence of a single dominant phage lineage [58, 59]. The phylogenetic diversity documented in this study mirrors the continuous evolutionary battle between phages and bacteria. Clinically, this diversity enables effective cocktail design. Phages from different lineages target distinct bacterial receptors through independent mechanisms, making simultaneous resistance mutations highly unlikely. This multi-lineage strategy now forms the foundation of personalized phage therapy against MDR *A. baumannii* [60, 61].

Pangenome analysis of the 340 virulent phage genomes yielded 20,982 unique protein families, of which 76.2% were cloud genes each detected in only a single genome, and no protein family was universally conserved at 100% amino acid identity across all 340 genomes. This highly open pangenome architecture, dominated by rare and lineage-specific gene content, strongly suggests that horizontal gene transfer and modular recombination are important contributors to genomic diversity in this phage population rather than vertical inheritance from a conserved ancestral gene repertoire. Open pangenomes of this scale have been increasingly documented in phage populations infecting genomically plastic bacterial hosts, where the flexible gene pool continuously generates novel phage variants through module exchange even when core genome sequences remain nearly identical [62]. Mobile genetic elements including phages are recognized as primary vehicles for horizontal gene transfer events that drive rapid microbial evolution, and their interactions with plasmids and chromosomal elements sustain open pan-genomic architectures across diverse microbial taxa [63]. The large reservoir of cloud proteins identified in this study almost certainly encodes novel anti-defense mechanisms, host metabolic manipulation factors, and structural adaptations with unknown biological functions. Their systematic experimental characterization represents one of the most promising frontiers for expanding our understanding of *Acinetobacter* phage biology beyond what sequence analysis alone can reveal.

Frequency-based protein analysis identified baseplate-associated components as the most recurrently detected protein class across the dataset, with multiple wedge, hub assembly, tail-tube junction, and puncturing device subunits detected in 48 to 50 phage genomes. The strong conservation of these components across phylogenetically divergent lineages reflects their indispensable role in infection. They function as the primary mediators of host surface contact, receptor recognition, and DNA injection trigger activation. A single spontaneous point mutation in the tail tubular protein of an *Acinetobacter* phage was sufficient to substantially expand its host range against MDR *A. baumannii* strains [23]. This confirms that tail components are the primary determinants of host specificity and that even minor structural variation can dramatically alter infection breadth. Together, these observations support the importance of tail-associated proteins in host recognition and suggest that variation in receptor-binding structures contributes substantially to host range diversity among *Acinetobacter* phages.

The alignment of *A. baumannii* CRISPR spacer sequences against the 340 phage genomes yielded 1,480 high-confidence matches distributed across 100 phage genomes, establishing that nearly one-third of the virulent phage population carries molecular evidence of prior infection of *A. baumannii* hosts. CRISPR spacers represent among the most reliable proxies for historical phage-host interactions, since each spacer is acquired de novo following a specific infection event and is retained in the host genome as a permanent immunological record of that encounter. An in-silico analysis of *A. baumannii* clinical genomes confirmed that CRISPR spacer sequences in this pathogen predominantly target bacteriophage sequences, supporting the potential use of spacer matching as an indicator of historical phage exposure in this specific host-pathogen system [37]. A large-scale survey of 4,977 *A. baumannii* RefSeq genomes found that approximately 13.84% of isolates carry functional CRISPR-Cas systems, with type I-F1 and I-F2 being the predominant subtypes and their co-existence significantly associated with enhanced restriction of phage invasion [36]. This means that the phages accumulating the highest spacer match counts in our dataset, including vB_AbaM_IME284, P11B, and HN_Aba_01 with 28 matches each, have demonstrably generated immune responses in CRISPR-active strains across diverse clinical backgrounds, providing strong molecular evidence for broad and recurrent historical infectivity. The approximately 70% of phages in our dataset lacking spacer matches should not be dismissed as therapeutically irrelevant. These phages may predominantly infect CRISPR-deficient strains or may encode efficient Anti-CRISPR mechanisms that suppress spacer acquisition; however, these possibilities were not directly evaluated in the present study. An analysis of CRISPR spacer content in 79 *A. baumannii* clinical isolates found that spacers associated with type I-F subtypes predominantly target prophage sequences rather than exogenous phages, suggesting that a significant fraction of acquired spacers reflects endogenous prophage regulation rather than active exogenous phage immunity [37]. Integrating CRISPR spacer evidence with phylogenetic and pangenome data provides a substantially richer picture of phage-host interaction history than any single line of evidence alone, and this multi-criteria approach is increasingly recognized as the standard for therapeutic phage candidate prioritization. While CRISPR spacer matching provides high-confidence evidence of historical phage-host interactions, alternative approaches including k-mer composition analysis, codon usage similarity, prophage homology searches, and machine learning-based host prediction methods are also available [64, 65]. Integrating these complementary strategies may improve host prediction coverage and resolution in future studies.

The guilt-by-association Anti-CRISPR identification pipeline revealed a highly skewed distribution of predicted Acr candidates, with *Acinetobacter* phage XC1 harboring 55 candidates, more than an order of magnitude greater than any other phage in the dataset. The genomic context of the XC1 candidates, specifically the clustering of twelve small hypothetical proteins within a contiguous genomic region flanked by a Gp33-like transcriptional regulator, is precisely consistent with canonical Acr locus architecture and strongly supports the prediction of genuine Anti-CRISPR function. The highest-scoring candidate, DMIAEELE_00254, achieved a composite support score of 3.0, satisfying all three predictive criteria of protein size, regulatory gene proximity, and cross-genome recurrence simultaneously. Previous studies have reported that Anti-CRISPR candidates are frequently associated with characteristics such as small protein size and proximity to Acr-associated regulatory genes [66]. Based on these observations, we incorporated protein size, regulatory gene proximity, and cross-genome recurrence as the three criteria in our composite scoring system. This genomic context–based strategy was selected because Anti-CRISPR proteins are highly diverse and frequently lack detectable sequence similarity to previously characterized Acr families, limiting the effectiveness of homology-based identification methods. Consequently, guilt-by-association approaches provide a practical framework for discovering putative novel Acr candidates that may be overlooked by sequence similarity searches alone.

A study examining Acr proteins in the context of bacterial antibiotic resistance demonstrated that phages encoding Acr proteins can successfully infect CRISPR-active strains while simultaneously facilitating horizontal gene transfer events between bacteria, a dual role that highlights both the therapeutic potential and the importance of conducting AMR gene screening in parallel with Acr identification [67]. Given that *A. baumannii* predominantly harbors type I-F CRISPR-Cas systems, consistent with observations across clinical isolate collections [68], these experimental insights are directly transferable to the therapeutic context addressed by the present study. More broadly, a class of RNA-based Anti-CRISPRs that sequester Cas proteins through mechanisms entirely distinct from protein-based inhibitors has recently been discovered, confirming that the landscape of CRISPR evasion strategies in phages is considerably broader than previously recognized [69]. The extraordinary Acr repertoire of *Acinetobacter* phage XC1 raises the possibility that this phage may encode multiple CRISPR-evasion mechanisms; however, experimental validation is required to confirm the functions of these predicted candidates. This may confer robust anti-immune capacity across different CRISPR-Cas subtypes present in *A. baumannii* clinical populations. The therapeutic utility of Anti-CRISPR proteins extends beyond enabling phage infection of CRISPR-protected hosts. These proteins can be incorporated into engineered phage platforms combining CRISPR-Cas-based targeting systems, which have shown promise for selectively eliminating antibiotic-resistant bacteria while sparing susceptible populations [17, 70]. This suggests that the Acr-rich genomic background of *Acinetobacter* phage XC1 could serve as both a therapeutic vehicle for overcoming bacterial CRISPR immunity and an engineering platform for incorporating programmable CRISPR-based targeting systems in next-generation phage-based interventions. However, the presence of a large Anti-CRISPR repertoire alone does not establish therapeutic suitability, and additional factors including host range, infection efficiency, environmental stability, safety, and in vivo efficacy must be experimentally evaluated before clinical application can be considered.

CARD screening identified several low-confidence homologs of AMR-associated proteins among the analyzed phage genomes. Because all matches were detected under the RGI Loose criterion and showed relatively low sequence identity to reference AMR proteins, no conclusions regarding functional antimicrobial resistance can be drawn from these data. Therefore, the AMR findings should be interpreted cautiously and primarily as indicators of distant sequence similarity. The *dfrA* and *qac* efflux gene homologs are similarly notable, as these families are linked to mobile resistance elements in clinical Gram-negative pathogens. However, all detections were Loose RGI hits below 50% amino acid identity to CARD references, indicating distant homology rather than confirmed resistance genes. As *dfrA* cassettes are commonly mobilized via class 1/2 integrons in clinical isolates [71], we screened all 71 *dfrA*-positive phages for integron architecture. No integrase genes (integron-specific or general phage) and no attC sites were found in any genome, and no complete integrons, CALIN, or In0 elements were identified. This absence, combined with the low sequence identity, indicates these *dfrA* homologs are not mobilized through canonical integron mechanisms and likely represent distant, non-functional homologs rather than clinically mobilized resistance genes. The presence of antibiotic resistance genes in phage genomes represents one of three commonly applied exclusion criteria for evaluating phage therapeutic suitability, alongside the capacity for generalized transduction and the presence of lysogeny markers [72]. Whole-genome sequencing enabling systematic AMR gene screening has been described as having transformed phage therapy from an empirical practice toward a standardized, personalized modality, and genomic safety infrastructure of the kind implemented here is the foundation upon which reproducible clinical phage therapy must be built [73]. Bacteriophage-mediated horizontal transfer of antibiotic resistance genes has been demonstrated to occur actively in natural and clinical microbial communities, with Caudovirales phages, which dominate our dataset, identified as particularly important vehicles for ARG transmission across bacterial populations [74]. The genomic role of phages in advancing and standardizing phage therapy applications requires that candidate selection frameworks explicitly integrate AMR screening rather than treating it as optional post-selection validation [75].

The finding that the majority of phage genomes in our dataset carry few or no AMR-associated genes is encouraging and confirms that therapeutically clean candidates are abundant within this collection. This pattern is consistent with phage-associated AMR gene findings across other Gram-negative pathogens including *Morganella morganii* and *Citrobacter freundii* [76, 77]. However, the heterogeneous distribution across the dataset makes individual-phage genomic screening indispensable and reinforces that phenotypic characterization alone is insufficient for responsible therapeutic candidate selection. The multi-criteria genomic framework developed in this study integrates genome composition profiling, phylogenetic characterization, pangenome analysis, CRISPR spacer matching, Anti-CRISPR identification, and AMR gene screening. This approach enables simultaneous optimization of therapeutic potential and safety in a way that single-criterion analyses cannot achieve. All analyses presented are in silico, and the prioritizations derived from genomic data require experimental validation through host range assays, bacterial killing experiments, and animal infection models before clinical relevance can be established. Future work should prioritize experimental characterization of high-priority candidates identified here, particularly those simultaneously combining high CRISPR spacer match counts, Acr enrichment, and clean AMR profiles. Longitudinal genomic surveillance of *Acinetobacter* phage populations in clinical environments will be essential. Ongoing antibiotic selection pressure continuously drives phage–host co-evolution, which may affect the relevance of prioritization frameworks derived from current datasets. The dataset and analytical pipeline established here provide a strong genomic foundation for integration with emerging phage engineering platforms. Such platforms enable targeted modification of receptor-binding proteins and systematic elimination of AMR gene content from candidate phages, directly addressing the safety and efficacy barriers that currently limit clinical translation. Together, these approaches hold particular promise for developing deployable interventions against carbapenem-resistant *A. baumannii*, which remains one of the most urgent unmet challenges in contemporary infectious disease medicine.

## Conclusion

Genomic analysis of 340 virulent *Acinetobacter* bacteriophages revealed extensive diversity in genome composition, phylogenetic architecture, and gene content organization. The phage population displayed an open pangenome dominated by accessory gene content, with no conserved core genome detectable at the sequence level, reflecting horizontal gene transfer and modular recombination as the primary evolutionary forces shaping this phage collection. CRISPR spacer matching and Anti-CRISPR profiling collectively identified phages with broad historical infectivity and robust anti-defense capacity, with *Acinetobacter* phage XC1 emerged as a high-priority candidate for future experimental evaluation owing to its unusually large repertoire of predicted Anti-CRISPR proteins. Systematic AMR screening confirmed the availability of genomically clean therapeutic candidates while flagging a subset carrying AMR gene homologs that warrant further verification before exclusion from clinical consideration. Collectively, these findings advance our understanding of *Acinetobacter* phage diversity and phage-host interaction dynamics, and establish a principled multi-criteria genomic framework for the rational prioritization of phage candidates against carbapenem-resistant *A. baumannii*. Further experimental validation of the high-priority candidates identified here holds significant promise for translating these genomic insights into effective phage-based interventions against multidrug-resistant Gram-negative infections.

## Supplementary Information

Below is the link to the electronic supplementary material.

Supplementary Material 1

Supplementary Material 2

Supplementary Material 3

Supplementary Material 4

Supplementary Material 5

Supplementary Material 6

**Supplementary Fig. 1** Distribution of predicted Anti-CRISPR candidates across representative Acr-enriched *Acinetobacter* phages. Candidate proteins were mapped according to their relative position within the annotated gene order of each genome. Red circles indicate predicted Anti-CRISPR candidates identified through the genomic context-based screening pipeline. Numbers on the right indicate the total number of candidates detected in each phage. Supplementary Material 7 (JPG 895 KB)

## Data Availability

No datasets were generated or analysed during the current study.

## Abbreviations

Aca: Anti-CRISPR-associated protein
Acr: Anti-CRISPR
AMR: Antimicrobial Resistance
ARG: Antimicrobial Resistance Gene
CRAB: Carbapenem-Resistant*Acinetobacter baumannii*
CRISPR: Clustered Regularly Interspaced Short Palindromic Repeats
MDR: Multidrug-Resistant
RGI: Resistance Gene Identifier
UPGMA: Unweighted Pair Group Method with Arithmetic Mean
VHDB: Virus-Host Database
XDR: Extensively Drug-Resistant

## Code availability

The Python scripts generated and used in this study for k-mer–based phylogenetic reconstruction, pangenome frequency analysis, protein frequency analysis, Anti-CRISPR candidate identification, and CRISPR spacer–based visualization are publicly available in a GitHub repository: https://github.com/imnayon/Acinetobacter_phage_analysis and archived in Zenodo: https://doi.org/10.5281/zenodo.20393043 to ensure reproducibility of the analyses.
