# Supplementary material for: Genomic landscape of 340 virulent Acinetobacter bacteriophages reveals anti-CRISPR–enriched candidates for therapeutic prioritization
Source: Eur J Clin Microbiol Infect Dis. 2026 Jul 6;45(10):3117–33. doi: 10.1007/s10096-026-05580-8 (PMC13676570; doi:10.1007/s10096-026-05580-8)
Supplement: Supplementary file 1 [file 10096_2026_5580_MOESM6_ESM.docx]

**Supplementary Table SX. IntegronFinder screening results for all dfrA-positive phage genomes.**

| **No.** | **Phage genome** | **dfrA allele** | **Genome length (bp)** | **Complete integrons** | **CALIN elements** | **In0 elements** | **Subthreshold attC hits** |
| --- | --- | --- | --- | --- | --- | --- | --- |
| 1 | Acinetobacter phage 133 | dfrA9 | 159,803 | 0 | 0 | 0 | 0 |
| 2 | Acinetobacter phage AB-Navy1 | dfrA35 | 166,113 | 0 | 0 | 0 | 0 |
| 3 | Acinetobacter phage AB-Navy4 | dfrA35 | 166,964 | 0 | 0 | 0 | 1 |
| 4 | Acinetobacter phage AB-Navy71 | dfrA9 | 166,382 | 0 | 0 | 0 | 0 |
| 5 | Acinetobacter phage AB-Navy97 | dfrA35 | 165,480 | 0 | 0 | 0 | 2 |
| 6 | Acinetobacter phage AB1I1M-1 | dfrA10 | 168,437 | 0 | 0 | 0 | 0 |
| 7 | Acinetobacter phage AB2I2 | dfrA10 | 61,679 | 0 | 0 | 0 | 0 |
| 8 | Acinetobacter phage AB2I3 | dfrA10 | 64,987 | 0 | 0 | 0 | 0 |
| 9 | Acinetobacter phage AC4 | dfrA35 | 168,186 | 0 | 0 | 0 | 1 |
| 10 | Acinetobacter phage AM101 | dfrA35 | 166,487 | 0 | 0 | 0 | 1 |
| 11 | Acinetobacter phage Ab3-Tpeters | dfrA38 | 95,782 | 0 | 0 | 0 | 0 |
| 12 | Acinetobacter phage Ab5 | dfrA38 | 96,601 | 0 | 0 | 0 | 0 |
| 13 | Acinetobacter phage Ab7 | dfrA38 | 96,601 | 0 | 0 | 0 | 0 |
| 14 | Acinetobacter phage AbTZA1 | dfrA9 | 168,223 | 0 | 0 | 0 | 0 |
| 15 | Acinetobacter phage Ab_121 | dfrA18 | 102,499 | 0 | 0 | 0 | 0 |
| 16 | Acinetobacter phage Abraxas | dfrA35 | 166,559 | 0 | 0 | 0 | 1 |
| 17 | Acinetobacter phage Ac42 | dfrA9 | 167,716 | 0 | 0 | 0 | 2 |
| 18 | Acinetobacter phage Acba_22 | dfrA18 | 104,980 | 0 | 0 | 0 | 0 |
| 19 | Acinetobacter phage Acj61 | dfrA9 | 164,093 | 0 | 0 | 0 | 0 |
| 20 | Acinetobacter phage Acj9 | dfrA9 | 169,947 | 0 | 0 | 0 | 0 |
| 21 | Acinetobacter phage Henu6 | dfrA9 | 166,890 | 0 | 0 | 0 | 0 |
| 22 | Acinetobacter phage KARL-1 | dfrA35 | 166,560 | 0 | 0 | 0 | 1 |
| 23 | Acinetobacter phage LemonAid | dfrA35 | 167,749 | 0 | 0 | 0 | 1 |
| 24 | Acinetobacter phage Liucustia | dfrA18 | 100,533 | 0 | 0 | 0 | 0 |
| 25 | Acinetobacter phage MD-2021a | dfrA35 | 164,311 | 0 | 0 | 0 | 1 |
| 26 | Acinetobacter phage Maestro | dfrA9 | 169,176 | 0 | 0 | 0 | 0 |
| 27 | Acinetobacter phage Melin | dfrA9 | 167,969 | 0 | 0 | 0 | 0 |
| 28 | Acinetobacter phage Meroveus | dfrA35 | 167,683 | 0 | 0 | 0 | 1 |
| 29 | Acinetobacter phage Minot | dfrA9 | 167,029 | 0 | 0 | 0 | 0 |
| 30 | Acinetobacter phage Mokit | dfrA9 | 167,638 | 0 | 0 | 0 | 0 |
| 31 | Acinetobacter phage Morttis | dfrA9 | 169,150 | 0 | 0 | 0 | 0 |
| 32 | Acinetobacter phage PhaR5 | dfrA35 | 164,549 | 0 | 0 | 0 | 1 |
| 33 | Acinetobacter phage Stupor | dfrA35 | 164,309 | 0 | 0 | 0 | 1 |
| 34 | Acinetobacter phage TAC1 | dfrA18 | 101,770 | 0 | 0 | 0 | 0 |
| 35 | Acinetobacter phage Tonic CPL00239 | dfrA35 | 168,103 | 0 | 0 | 0 | 0 |
| 36 | Acinetobacter phage UOL-KIMZ-24 | dfrA18 | 103,505 | 0 | 0 | 0 | 0 |
| 37 | Acinetobacter phage ZZ1 | dfrA9 | 166,687 | 0 | 0 | 0 | 0 |
| 38 | Acinetobacter phage phi1_092033 | dfrA18 | 104,070 | 0 | 0 | 0 | 0 |
| 39 | Acinetobacter phage phi1_092060 | dfrA35 | 167,208 | 0 | 0 | 0 | 2 |
| 40 | Acinetobacter phage vB_AbaM_14CRR8 | dfrA18 | 102,820 | 0 | 0 | 0 | 0 |
| 41 | Acinetobacter phage vB_AbaM_3014 | dfrA39 | 165,731 | 0 | 0 | 0 | 1 |
| 42 | Acinetobacter phage vB_AbaM_AB-Navy-v2 | dfrA35 | 167,167 | 0 | 0 | 0 | 0 |
| 43 | Acinetobacter phage vB_AbaM_Acibel004 | dfrA20 | 99,730 | 0 | 0 | 0 | 0 |
| 44 | Acinetobacter phage vB_AbaM_Apostate | dfrA35 | 164,746 | 0 | 0 | 0 | 1 |
| 45 | Acinetobacter phage vB_AbaM_B09_Aci01-1 | dfrA18 | 103,628 | 0 | 0 | 0 | 0 |
| 46 | Acinetobacter phage vB_AbaM_B09_Aci02-2 | dfrA18 | 104,354 | 0 | 0 | 0 | 0 |
| 47 | Acinetobacter phage vB_AbaM_B09_Aci05 | dfrA19 | 102,789 | 0 | 0 | 0 | 0 |
| 48 | Acinetobacter phage vB_AbaM_Berthold | dfrA35 | 165,927 | 0 | 0 | 0 | 0 |
| 49 | Acinetobacter phage vB_AbaM_CP14 | dfrA18 | 102,099 | 0 | 0 | 0 | 0 |
| 50 | Acinetobacter phage vB_AbaM_D22 | dfrA19 | 103,539 | 0 | 0 | 0 | 0 |
| 51 | Acinetobacter phage vB_AbaM_DLP1 | dfrA35 | 164,335 | 0 | 0 | 0 | 1 |
| 52 | Acinetobacter phage vB_AbaM_DLP2 | dfrA35 | 165,122 | 0 | 0 | 0 | 1 |
| 53 | Acinetobacter phage vB_AbaM_DLP3 | dfrA35 | 165,942 | 0 | 0 | 0 | 1 |
| 54 | Acinetobacter phage vB_AbaM_DP45 | dfrA35 | 164,031 | 0 | 0 | 0 | 1 |
| 55 | Acinetobacter phage vB_AbaM_Kimel | dfrA35 | 167,922 | 0 | 0 | 0 | 1 |
| 56 | Acinetobacter phage vB_AbaM_KissB | dfrA18 | 102,626 | 0 | 0 | 0 | 0 |
| 57 | Acinetobacter phage vB_AbaM_Konradin | dfrA35 | 166,210 | 0 | 0 | 0 | 1 |
| 58 | Acinetobacter phage vB_AbaM_Konradin-v2 | dfrA35 | 164,573 | 0 | 0 | 0 | 1 |
| 59 | Acinetobacter phage vB_AbaM_Lazarus | dfrA35 | 165,151 | 0 | 0 | 0 | 1 |
| 60 | Acinetobacter phage vB_AbaM_ME3 | dfrA35 | 234,900 | 0 | 0 | 0 | 0 |
| 61 | Acinetobacter phage vB_AbaM_Octan | dfrA35 | 166,760 | 0 | 0 | 0 | 1 |
| 62 | Acinetobacter phage vB_AbaM_P1 | dfrA18 | 107,537 | 0 | 0 | 0 | 2 |
| 63 | Acinetobacter phage vB_AbaM_PhT2 | dfrA9 | 169,554 | 0 | 0 | 0 | 0 |
| 64 | Acinetobacter phage vB_AbaM_PhT2-v2 | dfrA9 | 166,330 | 0 | 0 | 0 | 0 |
| 65 | Acinetobacter phage vB_AbaM_Rocket | dfrA18 | 104,232 | 0 | 0 | 0 | 1 |
| 66 | Acinetobacter phage vB_AbaM_phiAbaA1 | dfrA18 | 104,906 | 0 | 0 | 0 | 2 |
| 67 | Acinetobacter phage vB_AbaP_HB01 | dfrA18 | 112,623 | 0 | 0 | 0 | 1 |
| 68 | Acinetobacter phage vB_AbaS-Bhz15 | dfrA35 | 167,277 | 0 | 0 | 0 | 0 |
| 69 | Acinetobacter phage vB_AbaSI_3 | dfrA35 | 164,851 | 0 | 0 | 0 | 0 |
| 70 | Acinetobacter phage vB_AbaSt_W16 | dfrA9 | 166,741 | 0 | 0 | 0 | 0 |
| 71 | Acinetobacter phage vB_ApiM_fHyAci03 | dfrA35 | 165,975 | 0 | 0 | 0 | 1 |
